# Case Report: Adult fibrinous bronchitis associated with lymphatic reflux: a probable diagnosis based on convergent evidence

**DOI:** 10.3389/fmed.2026.1825038

**Published:** 2026-04-30

**Authors:** Huiwen Li, Lei Duan, Jianjian Yu, Chengsen Cai, Hao Wang, Jun Wang, Yuxia Liu

**Affiliations:** 1 College of First Clinical Medicine, Shandong University of Traditional Chinese Medicine, Jinan, Shandong, China; 2 Medical Affairs Department, The Second Affiliated Hospital of Shandong University of Traditional Chinese Medicine, Jinan, Shandong, China; 3 Department of Respiratory and Critical Care Medicine, The Second Affiliated Hospital of Shandong University of Traditional Chinese Medicine, Jinan, Shandong, China

**Keywords:** bronchial cast, fibrinous bronchitis, lymphatic reflux, misdiagnosis, plastic bronchitis

## Abstract

**Background:**

Fibrinous bronchitis, also known as plastic bronchitis, is a rare airway disorder characterized by branching bronchial casts that replicate the architecture of the bronchial tree. Adult cases are uncommon and frequently misdiagnosed because clinical manifestations and imaging findings are nonspecific. Diagnosis is particularly challenging in the absence of bronchoscopic or histologic confirmation of casts.

**Case presentation:**

We present a 52-year-old man with a 2-year history of recurrent cough and expectoration of branching mucoid casts. Serial chest imaging showed fluctuating infiltrates without bronchiectasis. Bronchoscopy did not identify casts, presumably because the procedure was performed outside the active expectoration phase. Microbiologic findings were intermittent and not associated with sustained response to antimicrobial therapy. Transbronchial biopsy demonstrated chronic mucosal inflammation with fibrinous exudation, consistent with a fibrin-predominant airway process. Radionuclide lymphoscintigraphy and direct lymphangiography showed mediastinal lymphatic dilatation and pulmonary lymphatic reflux. Based on a convergent evidence framework, a probable diagnosis of fibrinous bronchitis was made. This was based on reproducible cast morphology, supported by indirect histopathologic findings, objective lymphatic abnormalities, and the mismatch between symptoms and treatment response. The patient remained asymptomatic with no recurrence at 8 months following targeted lymphatic embolization.

**Discussion:**

This case highlights the diagnostic challenge of adult fibrinous bronchitis in the absence of direct bronchoscopic or histologic confirmation of casts. It suggests that reproducible morphologic features, when interpreted alongside supportive histologic findings and mechanism-oriented imaging, can provide a practical basis for diagnosis. Recognition of symptom–treatment discordance may further prompt reconsideration of non-infectious mechanisms, including lymphatic dysfunction.

**Conclusion:**

In the absence of direct confirmation, a probable diagnosis of fibrinous bronchitis can be supported by converging evidence. Reproducible morphologic patterns, integrated with mechanism-oriented imaging, offer a practical basis for diagnosis and management in rare airway diseases and may facilitate earlier recognition.

## 1 Introduction

Fibrinous bronchitis (FB), also known as plastic bronchitis (PB), is a rare airway disorder characterized by branching bronchial casts that can partially or completely obstruct the airways (1–3). Most published studies have focused on pediatric populations, particularly in association with congenital heart disease or postoperative lymphatic abnormalities (4, 5). To date, adult cases are limited to small case series and isolated reports (6), and the true incidence and clinical spectrum are not fully defined.

Diagnosis of FB in adults is challenging because symptoms and imaging findings are nonspecific and frequently overlap with infectious or inflammatory lung diseases (6, 7). Temporary responses to antimicrobial or anti-inflammatory therapy, coupled with microbiologic positivity, may reinforce initial diagnostic impressions. At the same time, cognitive biases like anchoring may delay recognition even more (8).

Recent advances in lymphatic imaging, including dynamic contrast-enhanced MR lymphangiography and intranodal lymphangiography, have revealed pulmonary lymphatic reflux and thoracic duct abnormalities in selected patients, providing a mechanistic explanation for cast formation (2, 9–11). Despite these insights, lymphatic evaluation is seldom incorporated early in adult patients presenting with recurrent “infection-like” episodes.

In this report, we present an adult patient with a 2-year history of recurrent airway symptoms whose diagnosis of FB was delayed. By reviewing the diagnostic trajectory, we highlight the importance of integrating bronchial cast morphology, lymphatic imaging, and clinical findings to improve recognition of adult FB and reduce diagnostic delay.

## 2 Case presentation

### 2.1 Patient information

**FIGURE 1:**
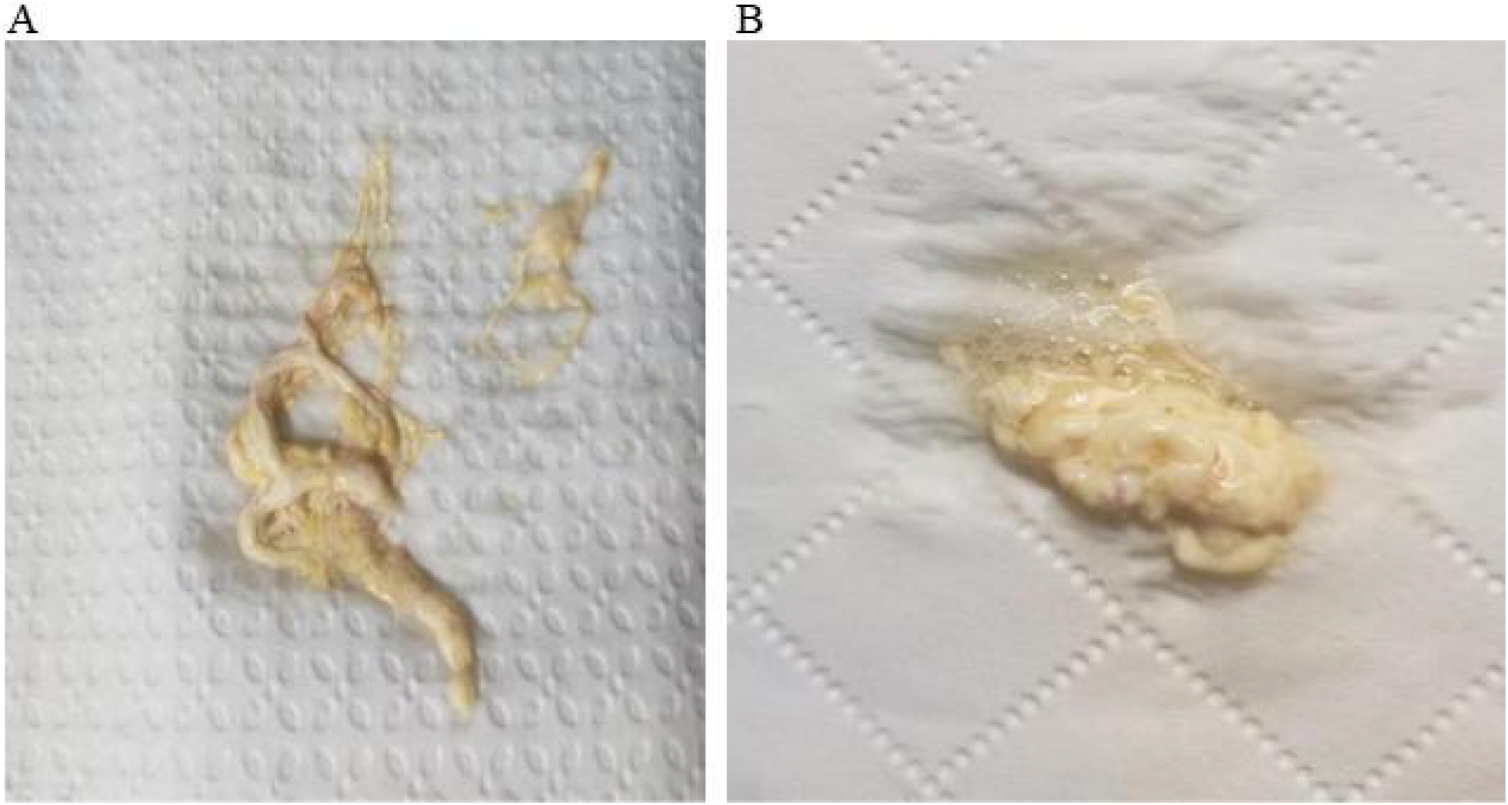
Bronchial cast expectorated by the patient **(A,B). (A,B)** Repeated expectoration of cohesive, branching cast-like material reproducing the segmental architecture of the bronchial tree. This morphology is highly suggestive of bronchial casts rather than ordinary mucus plugs and represents a key diagnostic clue in this case.

**FIGURE 2:**
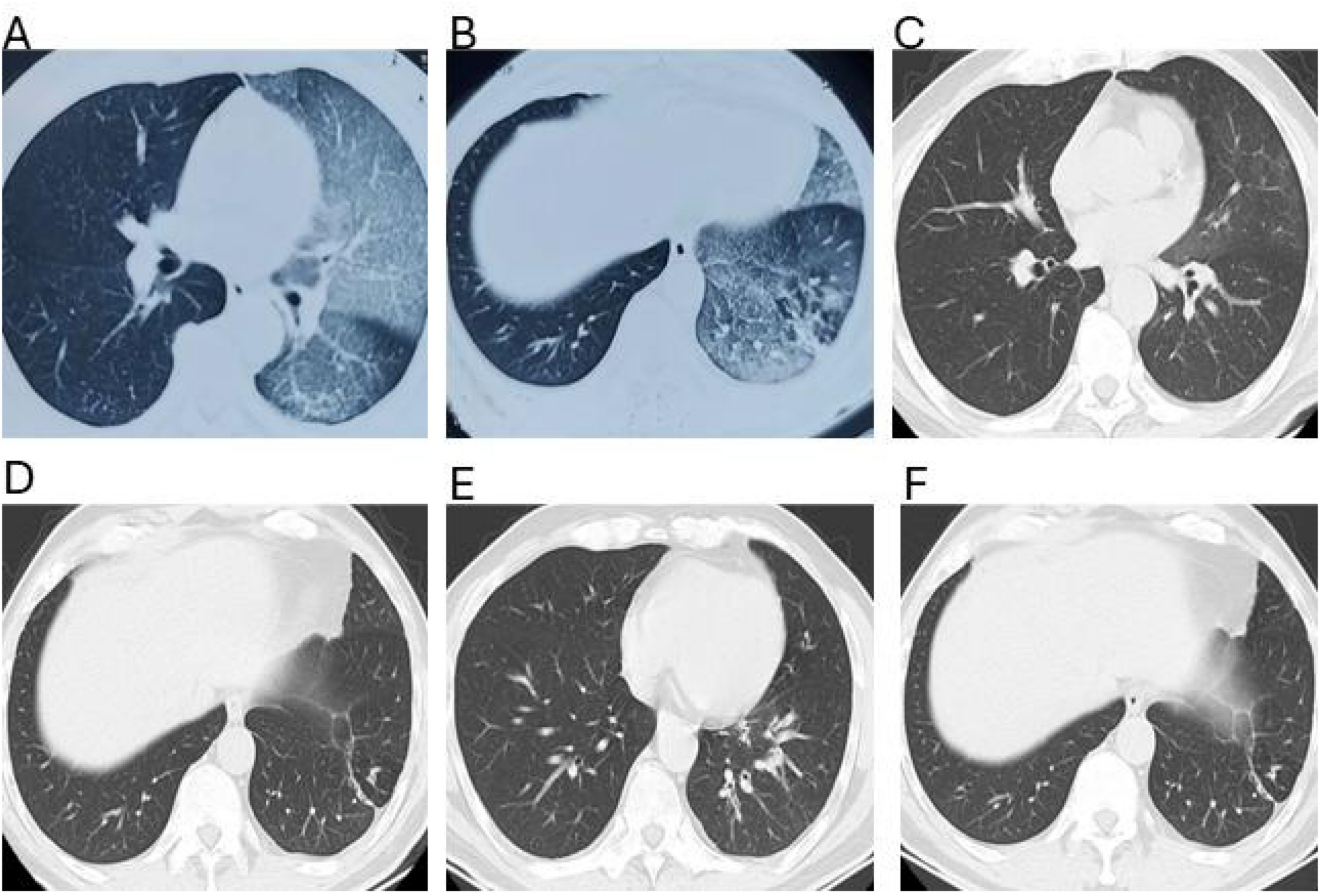
Serial chest computed tomography findings during the disease course **(A–F)**. **(A,B)** Chest CT performed in June 2023 demonstrates patchy consolidation and ground-glass opacities predominantly involving the lower lobe, with ill-defined margins. No bronchial dilation, wall thickening, or structural airway distortion is identified, suggesting an acute inflammatory or infectious process rather than chronic structural lung disease. **(C,D)** Chest CT obtained in March 2024 during symptom recurrence reveals new patchy and peribronchial opacities with a distribution different from the prior study. Partial resolution of previously involved areas is observed. The migratory pattern and absence of fixed anatomical abnormalities argue against bronchiectasis or persistent focal infection. **(E,F)** Chest CT performed in May 2024 shows scattered patchy infiltrates with interval fluctuation compared to earlier scans, with some lesions resolving and others newly appearing. Importantly, no irreversible bronchial dilation or airway remodeling is identified across serial imaging despite recurrent clinical episodes.

**FIGURE 3:**
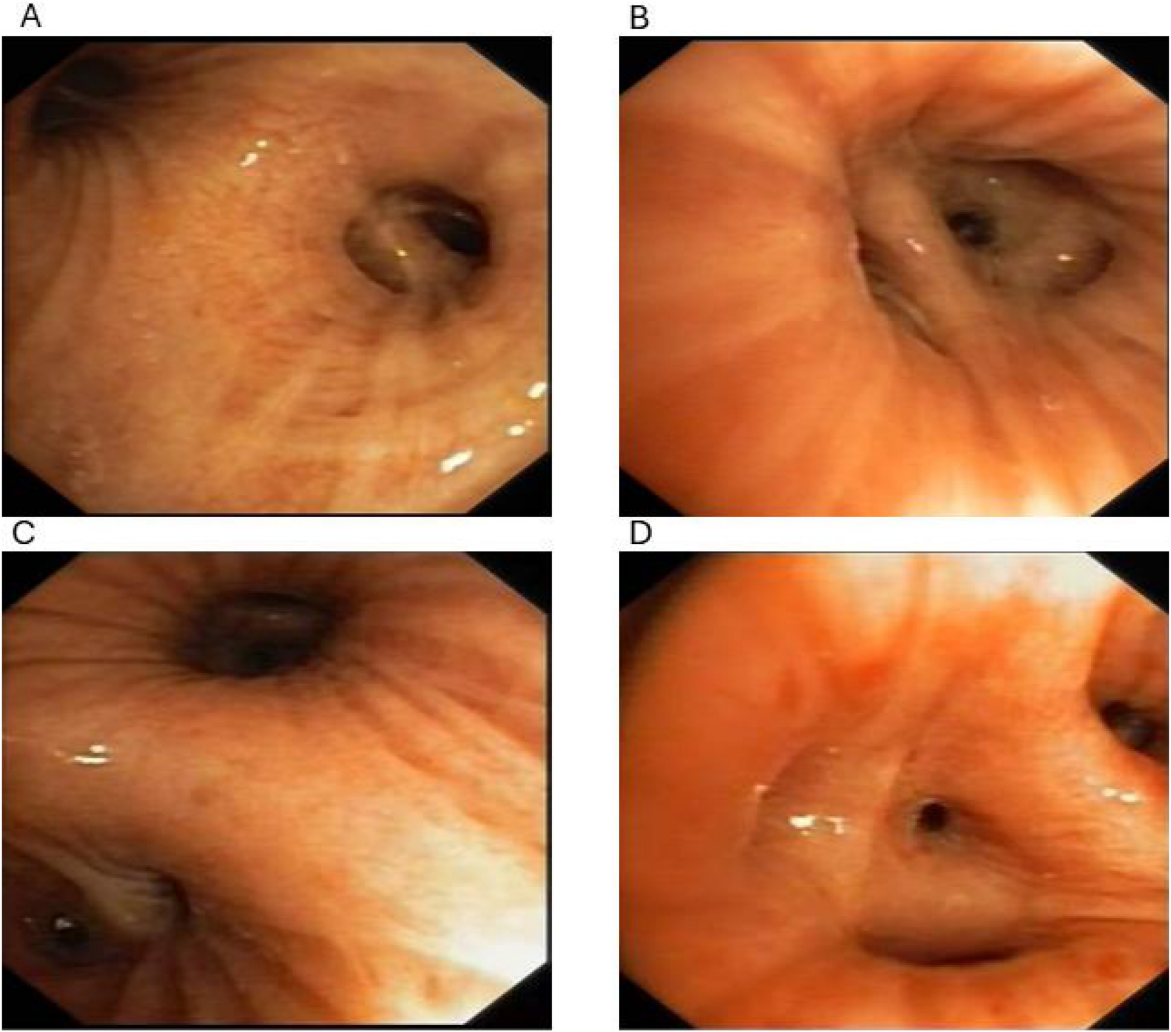
Bronchoscopic finding **(A–D). (A)** Left upper lobe segmental bronchus. The bronchial lumen is patent, with mild mucosal hyperemia and preserved longitudinal folds. No endobronchial mass, obstruction, or cast-like material is observed. **(B)** Opening of the basal segments of the left lower lobe. The bronchial orifice is mildly narrowed due to mucosal edema, with a small amount of clear secretions. No purulent discharge or obstructing lesion is identified. **(C)** Dorsal segment of the left lower lobe. The airway remains patent with mild mucosal swelling. No evidence of bronchial dilatation, intraluminal obstruction, or cast formation is observed. **(D)** Basal segment of the left lower lobe. Bronchial mucosa shows mild congestion and edema with slight luminal narrowing, but without visible endobronchial lesions or structured cast material. Overall, bronchoscopic examination demonstrates patent airways with nonspecific inflammatory changes, without evidence of obstructing casts, mass lesions, or purulent secretions. These findings argue against bronchiectasis or primary obstructive airway disease. The absence of visible casts may be explained by intermittent cast formation and expectoration prior to bronchoscopy or location of casts in distal airways beyond bronchoscopic reach. These bronchoscopic findings should be interpreted in conjunction with the overall clinical course, reproducible cast morphology, and lymphatic imaging abnormalities, which together support a probable diagnosis of fibrinous bronchitis.

**FIGURE 4:**
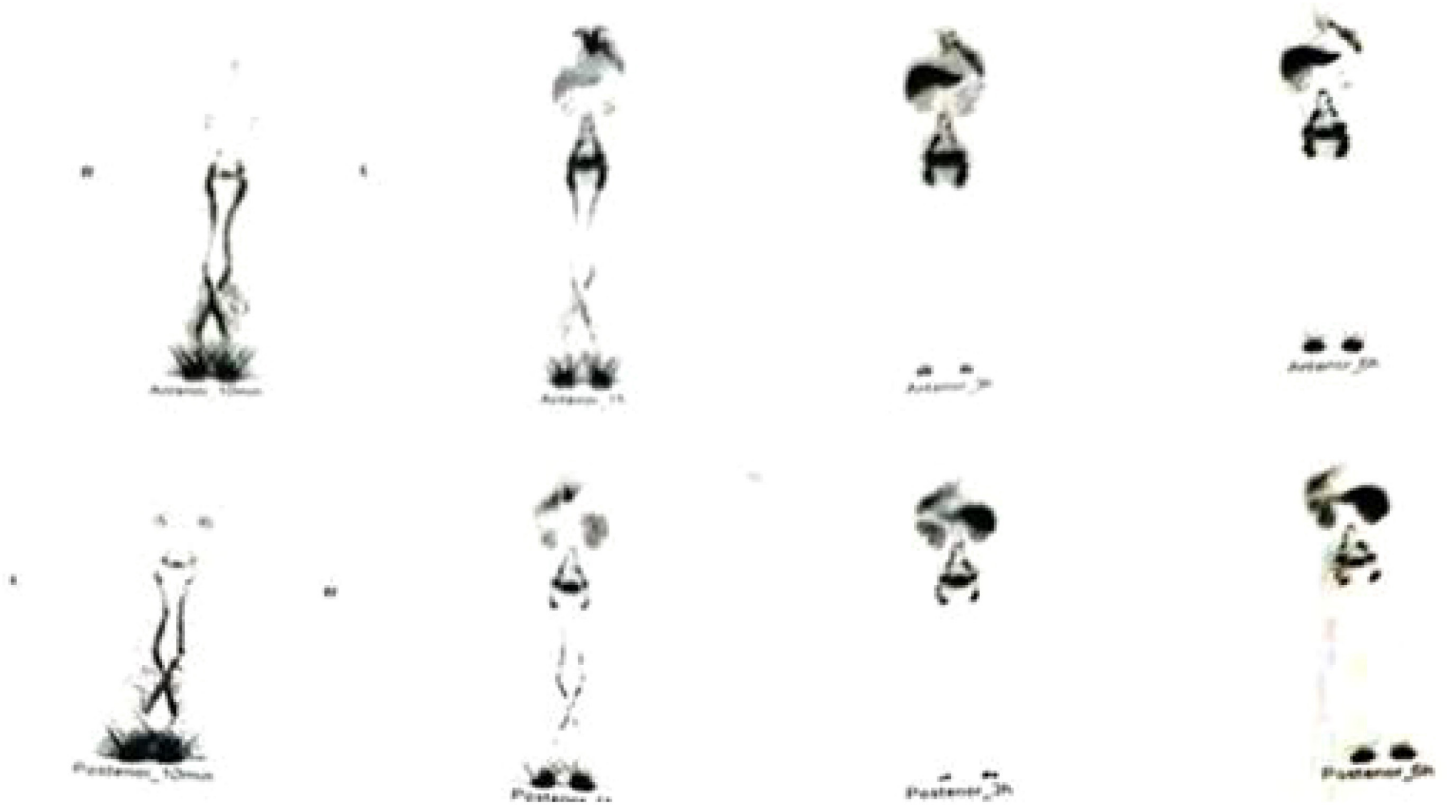
Whole-body radionuclide lymphoscintigraphy. Whole-body radionuclide lymphoscintigraphy demonstrating abnormal configuration of the thoracic duct with bilateral venous angle drainage and suspected outlet obstruction. Increased radiotracer accumulation is observed in the left thoracic cavity and pulmonary region, suggesting mediastinal–left hilar–left pulmonary lymphatic reflux. The resolution of symptoms following targeted lymphatic embolization further supports a potential mechanistic role of lymphatic dysfunction.

**FIGURE 5:**
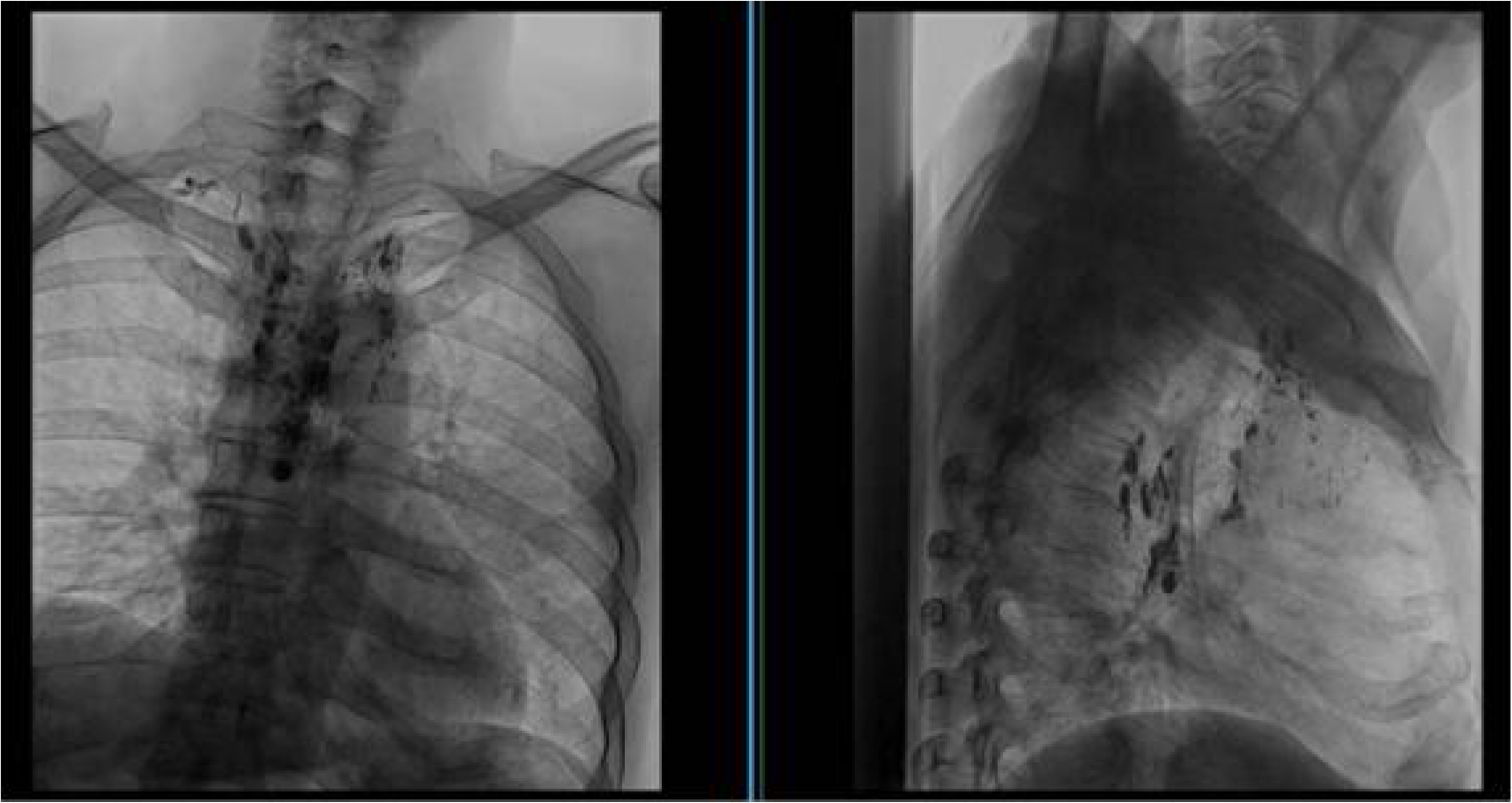
Fluoroscopic mediastinal lymphangiography. Fluoroscopic mediastinal lymphangiography demonstrating dilated lymphatic channels with contrast accumulation. Delayed imaging reveals irregular lymphatic vessels and contrast reflux within the mediastinum, indicating abnormal lymphatic flow dynamics. These abnormalities are consistent with previously reported mechanisms of lymphatic plastic bronchitis.

A 52-year-old man with a history of diabetes mellitus, hypertension, and coronary artery disease presented with recurrent cough and sputum production beginning in June 2023. He intermittently expectorated gray-white, branching, necrotic-appearing material (Figure 1). During the early course, these fragments were interpreted as necrotic debris or thick mucus plugs rather than bronchial casts. He denied persistent fever, chest pain, or significant dyspnea. No history of congenital heart disease or thoracic surgery was reported. The symptoms persisted for more than 2 years. Figures 1–5 illustrate the key diagnostic evidence, including morphologic features, nonspecific imaging findings, lymphatic abnormalities, and the clinical reasoning pathway leading to diagnostic reconsideration.

### 2.2 Diagnostic timeline

Over a period exceeding 2 years, the patient sought care at several tertiary hospitals. He was sequentially diagnosed with organizing pneumonia, infectious pneumonia, possible pulmonary sarcoidosis, and bronchiectasis with infection. At each stage, certain aspects of his clinical presentation or imaging findings appeared compatible with the working diagnosis, but none adequately explained the recurrent expectoration of branching material. The diagnostic trajectory and key decision points are summarized in Table 1, highlighting the evolving clinical reasoning and triggers for diagnostic reconsideration.

**TABLE 1:** Diagnostic timeline and key events.

Date	Working diagnosis	Key findings	Treatment	Outcome	Why reconsidered
2023.06	Organizing pneumonia	CT: inflammatory changes	Antibiotics + corticosteroids	Partial radiologic resolution; symptom relief	Recurrent symptoms; branching sputum unexplained
2024.01	Left lung inflammation	mNGS: *S. pneumoniae*, *E. coli*	Antibiotics	Mild improvement	No sustained response; structured sputum persists
2024.03	Possible sarcoidosis	Fluctuating CT findings	Corticosteroids ± antibiotics	Partial improvement; recurrence	No systemic evidence; casts unexplained
2024.05	Bronchiectasis with infection	CT: chronic inflammation; no fixed dilation	Adjusted steroids + antibiotics	Temporary improvement	No bronchial dilation; cast morphology atypical
2025.07	FB	Lymphatic imaging: reflux + duct abnormality	Lymphatic embolization	Sustained remission (8 months)	Integrated morphologic + imaging evidence

### 2.3 Detailed clinical course

#### 2.3.1 Initial phase (June 2023)

Chest CT revealed inflammatory changes in the left lung. Serial chest CT examinations performed during the subsequent disease course demonstrated fluctuating pulmonary abnormalities without a persistent obstructive lesion (Figures 2a,b). Organizing pneumonia was diagnosed, and the patient received multiple antibiotics combined with prednisone. Imaging showed significant improvement, and symptoms temporarily resolved.

#### 2.3.2 Reinforcement of infectious hypothesis (January 2024)

Symptoms recurred, with increased expectoration of necrotic-appearing material occasionally streaked with blood. Bronchoscopy with bronchoalveolar lavage (BAL) was performed in February 2024. Metagenomic next-generation sequencing (mNGS) of BAL fluid detected *Streptococcus pneumoniae* and *Escherichia coli*. Histopathologic examination of transbronchial biopsy from the left lingular segment demonstrated chronic mucosal inflammation with fibrinous exudation and infiltration of histiocytes. Immunohistochemistry showed CD68-positive histiocytes, while epithelial markers (CKpan) were negative. Special stains, including periodic acid–Schiff (PAS), acid-fast bacilli (AFB), and Grocott methenamine silver (GMS), were all negative. Although mNGS intermittently detected common respiratory pathogens, these findings were not consistent and did not correlate with sustained clinical response. Antimicrobial therapy resulted only in transient improvement, while branching cast expectoration persisted. Bronchoscopy was not performed during episodes of active cast expectoration, which may have reduced the likelihood of detecting intact bronchial casts. Overall, these findings suggest nonspecific inflammatory changes with possible secondary infection rather than a primary infectious process, prompting reconsideration of alternative diagnoses.

#### 2.3.3 Consideration of inflammatory lung disease (March 2024)

CT obtained on March 25, 2024 demonstrated pulmonary inflammatory changes (Figures 2C,D). Given the protracted course and fluctuating imaging findings, inflammatory lung disease was considered. Corticosteroid therapy again resulted in radiologic improvement, but symptoms did not fully resolve, and the diagnosis was reconsidered.

#### 2.3.4 Structural lung disease hypothesis (May 2024)

CT (Figures 2E,F) demonstrated features suggestive of chronic bronchitis and fibrotic changes in the left lower lobe. Repeat bronchoscopy in May 2024 showed patent airways in the right lung. In the left lung, mucosal hyperemia, edema, and segmental narrowing were observed, particularly in the basal segments of the lower lobe, with a small amount of thin secretions (Figure 3). Microbiologic evaluation of BAL fluid, including bacterial culture, fungal studies, and AFB smear, yielded negative or non-specific results. mNGS intermittently detected conditional pathogens, including Streptococcus constellation and Pseudomonas aeruginosa. A diagnosis of bronchiectasis with infection was favored. However, these findings were not consistently reproducible and did not correlate with sustained clinical response.

#### 2.3.5 Recognition of key morphologic clue (July 2025)

In July 2025, the patient presented to Beijing Hospital. Systematic review of the clinical history confirmed that the expectorated material consistently reproduced the branching architecture of the bronchial tree. Radionuclide lymphoscintigraphy (99mTc-DX) demonstrated abnormal lymphatic drainage characterized by thoracic duct malformation with bilateral venous angle drainage and suspected outlet obstruction (Figure 4). Increased radiotracer accumulation was observed in the left thoracic cavity, suggesting mediastinal–left hilar–left pulmonary lymphatic reflux (Figure 5). Cytologic examination (sputum-based thin-layer cytology) demonstrated predominantly lymphocytes.

Based on recurrent bronchial tree–like casts, discordance between clinical course and therapeutic response, and objective lymphatic abnormalities, a probable diagnosis of fibrinous bronchitis was made based on integrated clinical, morphologic, and lymphatic imaging findings. The patient underwent embolization of the responsible lymphatic vessels. The patient remained asymptomatic during 8 months of follow-up. Although follow-up duration remains relatively limited, no recurrence has been observed to date. Because no intact cast specimen was preserved for histologic analysis, the diagnosis was based on clinical, cytologic examination and lymphatic imaging findings.

### 2.4 Key ancillary findings

Comprehensive microbiologic evaluation was performed, including routine bacterial culture, fungal culture, and acid-fast bacilli (AFB) smear and mycobacterial culture, all of which were negative. Peripheral eosinophil count and total IgE were within normal limits. Pulmonary function testing revealed no obstructive or restrictive defects.

Bronchoalveolar lavage fluid (BALF) analysis showed a total cell count within normal limits, with a differential count demonstrating macrophages (∼90%), lymphocytes (∼8%), and neutrophils (∼2%), without eosinophilia. Cytologic examination, including sputum-based thin-layer cytology (TCT), demonstrated predominantly lymphocytes with a small number of squamous epithelial cells, and no evidence of malignant cells.

Comprehensive microbiologic evaluation was repeatedly negative or non-specific. mNGS intermittently detected common or opportunistic respiratory pathogens; however, these findings lacked consistency and were not associated with sustained clinical improvement following targeted antimicrobial therapy.

Collectively, these findings suggest that microbiologic positivity likely reflected colonization or secondary infection rather than a primary pathogenic driver, a known limitation in the clinical interpretation of mNGS results in respiratory specimens (12, 13).

Overall, despite extensive and repeated microbiologic investigations covering bacterial, fungal, and mycobacterial pathogens, no consistent or causative organism was identified. The cytologic profile, characterized by lymphocyte predominance without malignant features, further supports a non-neoplastic and likely non-specific inflammatory or lymphatic-related process.

### 2.5 Imaging interpretation

The chest CT (Figure 2) findings were nonspecific and primarily demonstrated inflammatory-appearing changes without a clear structural cause of airway obstruction. Lymphatic imaging (Figure 4) provided critical diagnostic information. Radionuclide lymphoscintigraphy revealed abnormal tracer accumulation within the mediastinum with reflux toward the pulmonary hilum. Direct lymphangiography further demonstrated dilated mediastinal lymphatic channels and abnormal retrograde lymphatic flow (Figure 5). These findings suggested the presence of a central lymphatic flow disorder, which has increasingly been recognized as an important mechanism in selected cases of plastic bronchitis (14, 15).

## 3 Discussion

### 3.1 Diagnostic challenges in adult FB

Adult FB remains underrecognized and is frequently misdiagnosed due to its nonspecific clinical and radiologic features. Unlike pediatric cases, which are frequently associated with congenital heart disease or postsurgical lymphatic abnormalities, adult cases often overlap symptomatically with more common infectious and inflammatory pulmonary disorders (5, 16–18). In this case, recurrent cough and fluctuating pulmonary infiltrates initially supported diagnoses such as infection or organizing pneumonia, particularly in the presence of transient treatment responses.

However, the persistent expectoration of branching material was not explained by these conditions. Early in the disease course, this finding was interpreted as nonspecific sputum, which contributed to diagnostic delay. This highlights a key challenge: distinctive morphologic features may be overlooked when they are not actively integrated into the diagnostic process.

### 3.2 Infection evidence with transient treatment response as a diagnostic warning pattern

Bronchiectasis with recurrent infection remains a plausible alternative explanation, particularly given intermittent microbiologic positivity and partial response to antimicrobial therapy (7, 19). However, several findings in this case are less consistent with bronchiectasis or a primary infectious process. Serial CT imaging demonstrated no irreversible bronchial dilatation, and the expectorated material consisted of cohesive, branching casts rather than purulent sputum, a feature not typical of bronchiectasis (20).

Despite repeated antibiotic courses, including at least one prolonged course ( ≥ 14 days) of antibiotics with appropriate spectrum coverage according to standard clinical guidelines for lower respiratory tract infection (21), sustained remission was not achieved. This pattern is consistent with prior reports indicating that antimicrobial therapy in plastic bronchitis often provides only transient benefit (22–24). Histopathologic findings demonstrating fibrinous exudation within the airway mucosa provide additional support for a non-infectious, fibrin-rich process rather than a purely infectious etiology. Although this finding was derived from transbronchial biopsy rather than an intact cast and is not specific, it offers indirect histologic support for the presence of a fibrinous airway process (25).

Rather than serving as a diagnostic discriminator alone, the pattern of transient response followed by relapse should be interpreted alongside persistent morphologic abnormalities, particularly reproducible cast formation. These findings suggest that infection, when present, is more likely secondary than primary. Taken together, this case illustrates a diagnostic pattern that may prompt reconsideration of alternative etiologies: (1) microbiologic positivity, (2) transient response to therapy, and (3) persistence of a distinctive morphologic abnormality. Identifying this pattern can help detect non-infectious mechanisms—including lymphatic dysfunction—earlier in adults with fibrinous bronchitis.

### 3.3 Morphologic evidence and its diagnostic value

Compared with previously reported adult cases in which diagnosis was confirmed by bronchoscopic extraction or histologic analysis of casts (7, 17), the present case lacks direct confirmation and therefore represents a lower level of diagnostic certainty. However, it illustrates that in selected scenarios, a probable diagnosis may still be established through converging indirect evidence (15, 26).

Among all findings, the repeated expectoration of casts reproducing the bronchial tree architecture represents the most distinctive and non-overlapping feature across differential diagnoses (27, 28). This pattern was consistent over time and not explained by alternative conditions.

The absence of bronchoscopic visualization warrants specific consideration. Cast formation in plastic bronchitis is often intermittent, and patients with an intact cough reflex may expectorate casts prior to bronchoscopy (22). In addition, casts located in distal airways may be beyond bronchoscopic reach, and prior treatment may contribute to partial dissolution (29). In this case, bronchoscopy was not performed during episodes of active cast expectoration, which likely reduced the probability of detecting intact casts and is consistent with prior reports describing intermittent cast formation and spontaneous expectoration before endoscopic evaluation (7, 25, 29).

Although histologic confirmation of cast composition was not obtained, transbronchial biopsy demonstrated fibrinous exudation within the airway mucosa. While not definitive, this finding provides supportive evidence linking morphologic observations to a fibrin-rich airway process.

Taken together, the diagnosis does not rely on any single element but on the consistency between reproducible morphology, supportive histology, and clinical course. This case also raises a broader diagnostic consideration: in rare airway disorders, reproducible morphologic features—particularly when supported by mechanism-driven imaging—may provide more reliable guidance than isolated or fluctuating microbiologic results. Implementing this approach in clinical practice could aid in earlier recognition and lower the risk of misdiagnosis for conditions that otherwise present with nonspecific clinical features.

### 3.4 Association with lymphatic abnormalities

The pathophysiological mechanism of adult fibrinous bronchitis remains incompletely understood. Increasing evidence suggests that lymphatic abnormalities may contribute to cast formation in a subset of patients (14, 15, 30). Advanced lymphatic imaging techniques, including dynamic contrast-enhanced MR lymphangiography and intranodal lymphangiography, have demonstrated abnormal pulmonary lymphatic reflux entering the airway in selected cases of plastic bronchitis (9, 31, 32). In the present case, multimodal lymphatic imaging demonstrated mediastinal lymphatic dilatation and pulmonary lymphatic reflux, consistent with previously described central lymphatic flow disorders (33–36). These findings support the hypothesis that abnormal lymphatic flow may allow protein-rich lymphatic fluid to enter the airway lumen, thereby contributing to cast formation (9, 15).

However, a direct causal relationship between lymphatic reflux and cast formation cannot be definitively established in the absence of histologic confirmation or interventional correlation. Previous studies and the present case have shown that selective lymphatic embolization can reduce or eliminate recurrent cast production in selected patients, supporting—though not universally proving—a mechanistic role of lymphatic flow disorders in plastic bronchitis (9, 10).

Therefore, in the present case, lymphatic dysfunction should be interpreted as a plausible contributory factor rather than a definitively proven mechanism.

### 3.5 A practical reappraisal strategy in suspected rare airway disorders

Collectively, this case shows that a clinically meaningful diagnosis of fibrinous bronchitis can still be made using converging lines of evidence, even in the absence of direct bronchoscopic or histologic confirmation. In this setting, reproducible cast morphology, supportive indirect histology, and lymphatic imaging findings are considered together rather than in isolation.

By addressing diagnostic uncertainty directly and systematically evaluating common alternative diagnoses, this approach helps move from uncertainty toward a more mechanism-based understanding of the disease. It also illustrates the practical value of integrating morphologic features, longitudinal clinical observations, and targeted imaging in patients with recurrent, unexplained airway symptoms.

Based on this case and the literature on diagnostic error and cognitive bias, we propose a pragmatic three-step reappraisal framework for patients with persistent, unexplained airway abnormalities: (1) Symptom–treatment discordance should prompt re-evaluation of the working diagnosis (37); (2) Distinctive morphologic findings, such as branching casts, should be documented and preserved when possible (22); (3) Mechanism-oriented investigations, including lymphatic imaging, should be considered when conventional explanations are insufficient (9).

This structured approach may serve as a cognitive safeguard against anchoring bias and facilitate earlier recognition of rare airway disorders, including adult plastic bronchitis.

### 3.6 Differential diagnosis

Table 2 summarizes a structured differential diagnostic analysis designed to systematically evaluate competing etiologies and delineate their key distinguishing features. Of the diagnostic considerations, bronchiectasis and organizing pneumonia represented the most plausible alternatives; nevertheless, neither entity fully accounted for the persistent, reproducible branching cast morphology observed in this patient. In addition, Allergic bronchopulmonary aspergillosis (ABPA) and other mucus plugging disorders were considered; however, the absence of eosinophilia, normal total IgE levels, and lack of supporting microbiologic or genetic evidence argued against these conditions. Sputum cytology did not demonstrate malignant cells, making an endobronchial neoplastic process unlikely.

**TABLE 2:** Differential diagnosis of recurrent branching sputum and rationale for exclusion in the present case.

Condition	Typical clinical/imaging features	Findings in this case	Reason for exclusion
Bronchiectasis with recurrent infection	Irreversible bronchial dilatation on CT; chronic productive cough with purulent sputum; recurrent infections with identifiable pathogens	Serial CT showed no irreversible bronchial dilatation; sputum was cohesive, branching casts rather than purulent sputum; microbiologic findings inconsistent	Lack of structural bronchial dilatation; morphology of expectorated material not typical; no sustained response to adequate antimicrobial therapy
Organizing pneumonia	Patchy or migratory pulmonary opacities; response to corticosteroids; relapse may occur	Transient radiologic improvement with corticosteroids; however, persistent expectoration of branching casts	Cast formation is not a recognized feature; persistent morphologic abnormality unexplained by organizing pneumonia
Allergic bronchopulmonary aspergillosis (ABPA)	Asthma history; eosinophilia; elevated total IgE; Aspergillus sensitization; central bronchiectasis	No history of asthma; no eosinophilia; normal total IgE; no microbiologic or immunologic evidence of Aspergillus	Absence of key immunologic and clinical criteria for ABPA
Chronic fungal or mycobacterial infection	Chronic symptoms; positive fungal cultures or AFB smear/culture; nodular or cavitary imaging findings	Repeated fungal cultures and AFB studies negative; imaging nonspecific without cavitation	No microbiologic confirmation; imaging not suggestive of chronic infection
Mucus plugging disorders (e.g., cystic fibrosis, CFTR-related disease)	Thick mucus plugging; often early-onset disease; associated with chronic sinusitis or genetic background	No childhood respiratory disease; no chronic sinusitis; no supportive clinical or genetic evidence	Clinical context not consistent; morphology of casts more structured and reproducible than typical mucus plugs
Endobronchial malignancy	Focal obstructive lesion; abnormal cytology; progressive symptoms	No endobronchial mass on imaging or bronchoscopy; cytology negative for malignancy	Lack of structural lesion and negative cytology

### 3.7 Limitations

This case has several limitations. Histologic analysis of intact casts was not performed, and therefore cast composition could not be definitively characterized. The diagnosis relies on indirect evidence and should be interpreted as probable. In addition, follow-up duration remains relatively limited.

## 4 Conclusion

Combined clinical, morphological, and lymphatic imaging findings support that this case is most consistent with lymphatic-associated bronchial cast disease, consistent with a probable diagnosis of fibrinous bronchitis.

This case provides three key contributions: (1) it demonstrates that a probable diagnosis of fibrinous bronchitis may be established based on converging indirect evidence in the absence of direct confirmation; (2) it highlights the diagnostic value of persistent morphologic abnormalities, particularly branching cast expectoration; and (3) it underscores the importance of lymphatic imaging in unexplained recurrent airway disease. Importantly, this case suggests that in the absence of direct bronchoscopic or histologic confirmation, a structured convergent-evidence approach may provide a practical diagnostic pathway for rare airway disorders.

## 5 Patient perspective

The patient described the 2-year course of illness as frustrating and confusing. He was particularly concerned about the repeated expectoration of unusual, branching sputum, which was not clearly explained during earlier evaluations. Although treatments occasionally brought temporary relief, the recurrence of symptoms led to ongoing uncertainty and anxiety about his condition.

After further assessment suggested a possible lymphatic cause, the patient reported a sense of relief in finally having a more coherent explanation. Following targeted treatment, his symptoms improved significantly, and no recurrence has been observed during follow-up. He expressed that achieving a clearer diagnosis and effective management greatly alleviated both his physical symptoms and psychological burden.

## Data Availability

The raw data supporting the conclusions of this article will be made available by the authors, without undue reservation.
